# The Role of Hedonic Goal Pursuit in Self-Control and Self-Regulation: Is Pleasure the Problem or Part of the Solution?

**DOI:** 10.1007/s42761-023-00193-2

**Published:** 2023-06-12

**Authors:** Daniela Becker, Katharina Bernecker

**Affiliations:** 1 Behavioural Science Institute, Radboud University, Thomas van Aquinostraat 4, 6525 GD Nijmegen, The Netherlands; 2 Department of Psychology, University of Zurich, Zurich, Switzerland; 3 URPP Dynamics of Healthy Aging, University of Zurich, Zurich, Switzerland

**Keywords:** Self-control, Self-regulation, Hedonic goals, Well-being, Affect regulation

## Abstract

This paper examines the role of hedonic goal pursuit in self-control and self-regulation. We argue that not all pursuit of immediate pleasure is problematic and that successful hedonic goal pursuit can be beneficial for long-term goal pursuit and for achieving positive self-regulatory outcomes, such as health and well-being. The following two key questions for future research are discussed: How can people’s positive affective experiences during hedonic goal pursuit be enhanced, and how exactly do those affective experiences contribute to self-regulatory outcomes? We also call for an intercultural perspective linking hedonic goal pursuit to self-regulatory outcomes at different levels. We suggest that understanding the cognitive, motivational, and affective mechanisms at play can help individuals reap the benefits of successful hedonic goal pursuit. Considering those potential benefits, hedonic goal pursuit should be studied more systematically. To achieve this, we argue for a stronger integration of affective science and self-control research.

Research on both self-control and self-regulation emphasizes the benefits of successful *long-term goal pursuit* (e.g., studying for an exam and dieting) for self-regulatory outcomes such as good health and well-being (de Ridder et al., 2012; Moffitt et al., 2011). In that landscape, *hedonic goals*, which promise the experience of immediate positive affect (e.g., enjoying the party and indulging in chocolate cake), are typically considered mere distractions, standing in the way of more important long-term goals. Even though hedonic goal pursuit and the experience of positive affect, pleasure, or enjoyment have been acknowledged as predictors of well-being and part of adaptive self-regulation more generally, most research on hedonic goals has focused almost exclusively on studying how to inhibit, avoid, or downregulate them (e.g., through employing self-control).

This critical attitude towards hedonic goal pursuit is pervasive in the self-control literature and beyond (Fitouchi et al., 2022). But is it justified? Here, we argue that not all pursuit of immediate pleasure is problematic. Sometimes it is (e.g., failing to inhibit an unwanted impulse); sometimes it is not (e.g., intentionally pursuing a hedonic goal; Becker & Bernecker, in press). This distinction is important, because as an emerging body of literature suggests, hedonic goal pursuit can also be a part of—rather than a threat to—successful long-term goal pursuit: For example, experiencing pleasure during food consumption is related to less rather than more food intake (Arch et al., 2018; Cornil & Chandon, 2016a, b). Making room for and enjoying off-work or off-study time supports rather than hinders performance (Grund et al., 2014; Jia et al., 2019; Sonnentag, 2018). And finally, people who are generally more successful at pursuing hedonic goals (high trait hedonic capacity) report not only more positive affect in their daily lives but also higher life satisfaction and fewer symptoms of depression and anxiety (Bernecker & Becker, 2021).

Together these findings call for a change in how we conceptualize and study the regulation of hedonic goals. They urge us to shift our focus from studying people’s *choices* (quantity) for either the long-term or hedonic goal (cake vs. fruit) to people’s affective *experience* (quality) while pursuing the chosen goal (enjoy eating cake or fruit). Whereas choices are often sufficient to book progress towards long-term goals (choosing the fruit to stay healthy), successful hedonic goal pursuit requires the experience of positive affect rather the mere choice (for the cake). In that respect, hedonic goals may resemble what has been termed emotion regulation goals, which are also about the up- or downregulation of a specific affective state (Tamir et al., 2019). Given the centrality of affective experiences, we advocate for a stronger integration of affective science and self-control research when addressing the following two questions: First, what supports and what hinders successful hedonic goal pursuit? Second, through what mechanism(s) does successful hedonic goal pursuit contribute to successful long-term goal pursuit and thereby to the attainment of desirable self-regulatory outcomes, such as good health, happy relationships, and career success?

## What Supports or Hinders Hedonic Goal Pursuit?

One central finding in the literature reviewed above is that the experience of pleasure and enjoyment does not always come easily. From a dual-process perspective (e.g., Strack & Deutsch, 2004), this may sound odd, given that the hedonic choice is usually portrayed as the easier choice, as it does not require the recruitment of control processes. However, recent research shows that people (i.e., especially those low in trait hedonic capacity) may have trouble actually enjoying a chosen hedonic activity (Bernecker & Becker, 2021). One of the factors undermining successful hedonic goal pursuit is the experience of intrusive thoughts about conflicting long-term goals (Bernecker & Becker, 2021; Grund et al., 2014; Shah & Kruglanski, 2002; Van der Wal & Van Dillen, 2013). Importantly, first evidence suggests that it is not so much about the successful inhibition of those intrusive thoughts, but more about whether they are spontaneously activated or not (Bernecker & Becker, 2021, Study 3).

Where do those intrusive thoughts come from? One common source are conflicting personal long-term goals or values. Even if people intentionally pursue a hedonic goal, they may experience intrusive thoughts because of the cost it has for progress on a conflicting long-term goal (e.g., thoughts about dieting spoil the pleasure of eating the chocolate cake). Moreover, given that people usually pursue multiple long-term goals (e.g., career success, health, and fitness), they may easily get the impression that any time or effort invested into a hedonic goal activity might be better invested into one of the many possible long-term goal activities. Such high potential of opportunity costs makes it difficult for people to tell apart situations in which hedonic goals may help or hurt self-regulatory outcomes. This in turn could lead to a general reluctance and inability to pursue hedonic goals.

Another less often studied source could be a culturally shaped critical attitude towards hedonic experiences. It has been argued that the tendency to moralize harmless pleasures and to praise restraint is present across cultures (Fitouchi et al., 2022). There are, however, also differences in the extent to which hedonic values are endorsed between countries (Saroglou et al., 2004; Schwartz & Sagie, 2000) and even between groups within one culture. Men, for example, find hedonism more important than women (Schwartz & Rubel, 2005), which fits common assumptions about gender roles (Harrington et al., 1992; Henderson & Dialeschki, 1991) and the finding that men have a higher trait hedonic capacity than women (Bernecker & Becker, 2021). Moreover, cultures may differ in their appreciation of hedonic goals and the ways in which hedonic behaviors are considered to be hedonically valuable vs. valuable for other purposes (e.g., eating hedonically pleasurable foods to celebrate cultural holidays or using substances for spiritual purposes). Indeed, the ethical question arises as to whether it is appropriate to intervene on hedonic behaviors—even if they may have long-term negative consequences—when that behavior is an important component of cultural practices.

In the future, it will be interesting to test whether people’s belief that engaging in hedonic behaviors is “wrong” is related to more intrusive thoughts and feelings of guilt during hedonic goal pursuit and also whether those beliefs undermine the potential benefits hedonic goal pursuit may have for self-regulation. If so, one way to reduce intrusive thoughts could be adopting a less critical attitude towards hedonic goal pursuit. Mindfulness may be a promising candidate here for two reasons: mindfulness enhances present moment awareness and involves adopting a non-judgmental attitude towards upcoming thoughts. To date, most research has focused on how mindfulness helps regulate negative affect, but there is also evidence that it may also help savor hedonic experiences (e.g., Garland, 2021). Another way to make hedonic goal pursuit more successful may be the use of pro-active strategies. According to recent developments in the self-control literature, which in turn were inspired by emotion regulation research, self-control is not only about successful inhibition but also (or even more so) about the use of strategies that help avoid conflict in the first place (Duckworth et al., 2016; Hennecke & Bürgler, 2020). For example, one could change the situation so that intrusive thoughts are less likely to arise (e.g., switching off the phone during evening off) or reappraise hedonic goal pursuit as beneficial. Future research will tell if those strategies can support not only long-term but also hedonic goal pursuit.

## How Does Hedonic Goal Pursuit Contribute to Long-term Goal Pursuit and Self-Regulatory Outcomes?

We argued above that hedonic goal pursuit is part of self-regulation and may support the attainment of long-term goals. But how? One line of research focuses on *coupling* hedonic experiences to long-term goal pursuit, so that the latter becomes more appealing and will be engaged in more successfully and/or more frequently. This has been studied by, for example, highlighting the pleasurable aspects of a long-term goal activity (e.g., exercising is fun; Woolley & Fishbach, 2016; see also Shiota et al., 2021), or by encouraging people to link hedonic activities to long-term goal activities (e.g., students may bring their favorite milkshake to the library; Milkman et al., 2014). In those studies it remains, however, unclear whether participants were primarily pursuing a hedonic or long-term goal—or both as a function of the manipulation. Another way of coupling two competing goals is rewarding oneself with a hedonic activity after successful long-term goal pursuit (e.g., deserving the evening off after a long work week; Hennecke & Bürgler, 2020; Jia et al., 2019).

In those approaches, hedonic goals stand in the *direct* service of long-term goals. In that way, hedonic goals become more like a mean supporting long-term goal pursuit rather than a goal by itself. But is that the only way in which hedonic goals support long-term goal pursuit, or is enjoying the chocolate cake in and of itself beneficial in any way, too? In other words, should we encourage people to do strategic coupling, or should we also teach them to enjoy the occasional hedonic activity for the sake of enjoyment alone? What speaks for the latter is that hedonic goals, if pursued successfully, are characterized by positive affect, which not only enhances well-being in general (Bernecker & Becker, 2021), but which also facilitates the initiation of (especially difficult) goal-directed action (Kuhl et al., 2021; Taquet et al., 2016). Moreover, including hedonic “deviations” into a goal-striving plan has been shown to increase and sustain overall motivation without costs for long-term goal pursuit (Coelho do Vale et al., 2016; Prinsen et al., 2018). Finally, work in the eating domain suggests that the enjoyment people experience while eating may help regulate food intake (e.g., smaller meal size and reduced subsequent snacking; Arch et al., 2018; Cornil & Chandon, 2016a, b) through enhancing attention, the sense of appreciation, and satisfaction. These findings are in line with research suggesting that it is the obstructed enjoyment (e.g., through distractions) that motivates people to compensate, through either increasing intensity or quantity (van der Wal & van Dillen, 2013).

Different from the approaches discussed above, here, hedonic goals stand in the *indirect* service of a long-term goal, because the primary goal is to enjoy the meal, which reduces the amount people eat as side effect. An interesting question for future research is, therefore, to test whether the satisfaction that can be obtained through undiluted enjoyment may protect people from overconsumption—and if so, how Taken together, the successful pursuit of hedonic goals and the resulting experience of positive affect and enjoyment have been shown to support long-term goals directly and indirectly. In the coming years, more systematic research is, however, necessary to map the different ways through which hedonic experiences contribute to self-regulatory outcomes.

## Limitations and Discussion

An emerging literature suggests that hedonic goal pursuit is part of adaptive self-control and self-regulation more generally. In order to reap the benefits of successful hedonic goal pursuit, we need to gain a better understanding of the cognitive, motivational, and affective mechanisms at play. We proposed two key questions for future research on the affective mechanisms: How can people’s positive affective experiences during hedonic goal pursuit be enhanced, and how exactly do those experiences contribute to long-term goal pursuit and obtaining desirable self-regulatory outcomes? Given the emphasis on the affective *experience* during hedonic goal pursuit (rather than on people’s choices), we propose a stronger integration of affective and self-regulatory science to successfully answer those questions.

As mentioned above, cultures and countries differ in the degree to which they endorse hedonic values. Some argue that we already live in a culture in which pleasure is overly endorsed (Baumeister & Heatherton, 1996), while others see hedonic behaviors overly moralized (Fitouchi et al., 2022). Self-regulation research, however, is primarily conducted in Western, educated, industrialized, rich, and democratic (WEIRD) samples by WEIRD researchers. What is needed is an intercultural perspective linking hedonic goal pursuit to self-regulatory outcomes at different levels (cf. Kamiloğlu et al., 2020; Sauter et al., 2009). We call for psychological research to examine (in an unbiased way) how the *quality* of hedonic experiences can be improved and which levels of *quantity* are ideal for individuals’ and societies’ long-term interests.

## References

[CR1] Arch JJ, Warren K, Goodman RJ, Della MD, Kiken LG, Tillman S (2018). Enjoying food without caloric cost: The impact of brief mindfulness on laboratory eating outcomes. Behaviour Research and Therapy.

[CR2] Baumeister RF, Heatherton TF (1996). Self-regulation failure: An overview. Psychological Inquiry.

[CR3] Bernecker K, Becker D (2021). Beyond self-control: Mechanisms of hedonic goal pursuit and its relevance for well-being. Personality and Social Psychology Bulletin.

[CR4] Coelho do Vale R, Pieters R, Zeelenberg M (2016). The benefits of behaving badly on occasion: Successful regulation by planned hedonic deviations. Journal of Consumer Psychology.

[CR5] Cornil Y, Chandon P (2016). Pleasure as a substitute for size: How multisensory imagery can make people happier with smaller food portions. Journal of Marketing Research.

[CR6] Cornil Y, Chandon P (2016). Pleasure as an ally of healthy eating? Contrasting visceral and Epicurean eating pleasure and their association with portion size preferences and wellbeing. Appetite.

[CR7] de Ridder DTD, Lensvelt-Mulders G, Finkenauer C, Stok FM, Baumeister RF (2012). Taking stock of self-control: A meta-analysis of how trait self-control relates to a wide range of behaviors. Personality and Social Psychology Review.

[CR8] Duckworth AL, Gendler TS, Gross JJ (2016). Situational strategies for self-control. Perspectives on Psychological Science.

[CR9] Fitouchi, L., André, J.-B., & Baumard, N. (2022). Moral disciplining: The cognitive and evolutionary foundations of puritanical morality. *Behavioral and Brain Sciences, 1–71*. 10.1017/s0140525x22002047 10.1017/S0140525X22002047 36111617

[CR10] Garland EL (2021). Mindful positive emotion regulation as a treatment for addiction: From hedonic pleasure to self-transcendent meaning. Current Opinion in Behavioral Sciences.

[CR11] Grund A, Brassler NK, Fries S (2014). Torn between study and leisure: How motivational conflicts relate to students’ academic and social adaptation. Journal of Educational Psychology.

[CR12] Harrington M, Dawson D, Bolla P (1992). Objective and subjective constraints on women’s enjoyment of leisure. Society and Leisure.

[CR13] Henderson KA, Dialeschki MD (1991). A sense of entitlement to leisure as constraint and empowerment for women. Leisure Sciences.

[CR14] Hennecke M, Bürgler S (2020). Many roads lead to Rome self-regulatory strategies and their effects on.pdf. *Soc Personal Psychol*. Compass.

[CR15] Jia L, Hirt ER, Koh AHQ (2019). How to have your cake and eat it too: Strategic indulgence in big-time collegiate sports among academically successful students. Social Psychological and Personality Science.

[CR16] Kamiloğlu RG, Fischer AH, Sauter DA (2020). Good vibrations: A review of vocal expressions of positive emotions. Psychon Bull Rev.

[CR17] Kuhl, J., Quirin, M., & Koole, S. L. (2021). *The functional architecture of human motivation: personality systems interactions theory*. 1–62. 10.1016/bs.adms.2020.06.001

[CR18] Milkman KL, Minson JA, Volpp KGM, Milkman KL (2014). Holding the hunger games hostage at the gym: An evaluation of temptation bundling. Management Science.

[CR19] Moffitt TE, Arseneault L, Belsky D, Dickson N, Hancox RJ, Harrington H, Houts R, Poulton R, Roberts BW, Ross S, Sears MR, Thomson WM, Caspi A (2011). A gradient of childhood self-control predicts health, wealth, and public safety. Proceedings of the National Academy of Sciences of the United States of America.

[CR20] Prinsen S, Evers C, Wijngaards L, Van Vliet R, Ridder DD (2018). Does self-licensing benefit self- regulation over time ? An ecological momentary assessment study of food temptations. Personality and Social Psychology Bulletin.

[CR21] Saroglou V, Delpierre V, Dernelle R (2004). Values and religiosity: A meta-analysis of studies using Schwartz’s model. Personality and Individual Difference.

[CR22] Sauter, D. A., Eisner, F., Ekman, P., & Scott, S. K. (2009). Cross-cultural recognition of basic emotions through nonverbal emotional vocalizations. 10.1073/pnas.0908239106 10.1073/pnas.0908239106 PMC2823868 20133790

[CR23] Schwartz SH, Rubel T (2005). Sex differences in value priorities: Cross-cultural and multimethod studies. Journal of Personality and Social Psychology.

[CR24] Schwartz SH, Sagie G (2000). Value consensus and importance: A cross-national study. Journal of Cross-Cultural Psychology.

[CR25] Shah JY, Kruglanski AW (2002). Priming against your will: how goal pursuit is affected by accessible alternatives. Journal of Experimental Social Psychology.

[CR26] Shiota MN, Papies EK, Preston SD, Sauter DA (2021). Positive affect and behavior change. Current Opinion in Behavioral Sciences.

[CR27] Sonnentag S (2018). The recovery paradox: Portraying the complex interplay between job stressors, lack of recovery, and poor well-being. Research in Organizational Behavior.

[CR28] Strack F, Deutsch R (2004). Reflective and impulsive determinants of social behavior. Personality and Social Psychology Review : An Official Journal of the Society for Personality and Social Psychology, Inc.

[CR29] Tamir M, Halperin E, Porat R, Bigman YE, Hasson Y (2019). When there’s a will, there’s a way: Disentangling the effects of goals and means in emotion regulation. Journal of Personality and Social Psychology.

[CR30] Taquet M, Quoidbach J, de Montjoye Y-A, Desseilles M, Gross JJ (2016). Hedonism and the choice of everyday activities. Proceedings of the National Academy of Sciences.

[CR31] Van der Wal RC, Van Dillen LF (2013). Leaving a flat taste in your mouth: task load reduces taste perception. Psychological Science.

[CR32] Woolley K, Fishbach A (2016). For the fun of it: Harnessing immediate rewards to increase persistence in long-term goals. Journal of Consumer Research.

